# Spatial ecology of two emblematic deep-sea crustaceans in the Salas y Gómez, Nazca and Juan Fernández ridges Southeast Pacific

**DOI:** 10.1038/s41598-025-98820-4

**Published:** 2025-04-25

**Authors:** Maximiliano Fernández-Zúñiga, Rodolfo Vögler, María de los Ángeles Gallardo, Jan M. Tapia-Guerra, Javier Sellanes

**Affiliations:** 1 Departamento de Biología Marina, Facultad de Ciencias del Mar, Universidad Católica del Norte, sede Coquimbo, Coquimbo, Chile; 2 Departamento de Modelización Estadística de Datos e Inteligencia Artificial, Centro Universitario Regional del Este- Sede Rocha (CURE-Rocha), Universidad de la República, Rocha, Uruguay; 3 Centro de Ecología y Manejo Sustentable de Islas Oceánicas, Facultad de Ciencias del Mar, Universidad Católica del Norte, Coquimbo, Chile; 4 Programa de Doctorado en Biología y Ecología Aplicada, Universidad Católica del Norte, Larrondo 1281, Coquimbo, Chile; 5 Sala de Colecciones Biológicas, Facultad de Ciencias del Mar, Universidad Católica del Norte, Coquimbo, Chile

**Keywords:** Habitat characterization, Remote high-seas ecosystems, Endemic crustacean species, Marine protected areas, Seamounts, Biogeography, Ecosystem ecology, Biogeography, Ecological modelling, Ecosystem ecology

## Abstract

The Salas & Gómez, Nazca, and Juan Fernández ridges are among the least studied regions on the planet. Ecological knowledge of deep-sea organisms inhabiting islands and seamounts along these ridges is limited. Using various sources, including published data and in situ information from five scientific expeditions in the region, we analyzed the spatial ecology of *Paromola rathbuni* and *Projasus bahamondei*, two mobile species of benthic megafauna. Random Forest models detected different combinations of abiotic variables affecting species distribution. The distribution of *P. bahamondei* was primarily influenced by dissolved oxygen, longitude (W), and temperature. The distribution of *P. rathbuni* was mainly explained by longitude (W), followed by depth, temperature, and oxygen. Generalized Linear Models quantified significant effects of low dissolved oxygen and temperature on species presence, influencing their preference for bathymetric ranges (*P. bahamondei* at 400–500 m, *P. rathbuni* at 300–400 m). A distribution break to the west of ~ 85°W was confirmed, potentially due to the weakening of the Oxygen Minimum Zone altering oxygen and thermal conditions. This feature could act as a barrier for both species despite their high dispersal potential. Under projected climate change, shifts in latitudinal, longitudinal, and bathymetric distribution patterns are expected for both species.

**Supplementary Information:**

The online version contains supplementary material available at 10.1038/s41598-025-98820-4.

## Introduction

In the southeast Pacific (SEP), Salas & Gómez (S&G), Nazca (NZ), and Juan Fernández (JF) ridges are globally recognized as a biodiversity hotspots that high biological and ecological value enhanced by their role as trans-Pacific corridors for numerous migratory species and as nursery areas^1–4^. Because of the S&G and NZ ridges unique biodiversity, having the highest levels of endemism known for marine ecosystems and the deepest light-dependent reefs in the world^4,5^, the Convention on Biological Diversity (CBD) declared this remote high seas region as an Ecologically or Biologically Significant Marine Area (EBSA) in 2014^4,6^. Despite their importance these ridges remain among the least studied regions of the Pacific due to their isolation and the high costs associated with deep-sea research^2,4,7^.

The S&G and NZ ridges together extend nearly 3,000 km and comprise more than 110 seamounts, whose summits range from 150 to ~ 1,000 m below the surface. These ridges are located in an ultraoligotrophic region, where they represent a biodiversity oases due to the heterogeneity of their geomorphology, which enhances recirculation and local upwelling^3,8,9^. Along the ridges, oceanographic conditions (temperature, salinity, and oxygen) vary both longitudinally and bathymetrically^8^. The NZ and JF ridges, as well as the intersection of S&G and NZ, are influenced by the oceanic portion of the Humboldt Current System (HCS), which is characterized by cold subsurface waters, low oxygen levels, and high nutrient content that are typical conditions of the Oxygen Minimum Zone (OMZ)^8,10^. The western limits of the HCS influence are estimated to be between 82° and 84°W, coinciding with the biogeographic transition zone proposed by Parin et al.^11^. Further west, the influence of subtropical waters increases, leading to higher temperatures and dissolved oxygen levels but lower nutrient concentrations^2,8^. The longitudinal and bathymetric gradients of oceanographic conditions may influence community structure along the ridges^9,12^. For example, the OMZ represents a barrier to the distribution of many benthic megafauna taxa that are intolerant to hypoxic conditions^13–16^, whereas species that tolerate hypoxic conditions exhibit physiological and morphological adaptations that enable them to perform their metabolic and biological activities in low-oxygen environments^13,17^.

Three large decapod crustacean species are common in the mesophotic benthic communities of S&G, NZ, and JF ridges and Desventuradas Islands (east of S&G and NZ intersection): *Projasus bahamondei* (Decapoda: Achelata), *Paromola rathbuni* (Decapoda: Brachyura), and *Chaceon chilensis* (Decapoda: Brachyura)^9,11,18–20^. *Projasus bahamondei* (Juan Fernández king crab) and *P. rathbuni* (Chilean jagged lobster) are part of the bycatch fauna in the artisanal *C. chilensis* fishery, which is carried out using crustacean traps in the Desventuradas Islands and the JF Archipelago^18,19^. *Paromola rathbuni* is consumed locally and thus is a secondary interest of artisanal fisheries. In contrast, *P. bahamondei* has no local market, but its abundance and frequency of bycatch have promoted exploratory fishing studies for other markets^19^.

There is scarce biogeographic and ecological information (e.g., intra- and interspecific relationships, habitat use) on the benthic communities inhabiting the mesophotic zone of the S&G, NZ, and JF ridges, particularly for *P. bahamondei* and *P. rathbuni*. Moreover, their easy identification, available fishery data, and absence in the S&G ridge make these large decapods a useful model for assessing which environmental variables could explain the discontinuity in their longitudinal distribution.

Therefore, the aim of our study is to analyze the spatial distribution and ecology of *P. bahamondei* and *P. rathbuni* along the S&G, NZ, and JF ridges. Specifically, we consider two objectives: (i) to model species-environment relationships along the horizontal (longitudinal) and vertical (bathymetric) axes, estimating whether a set of oceanographic variables (temperature, salinity, and dissolved oxygen) could affect the distribution of these species and (ii) to describe the ecology of both species, including their habitat use, habitat characteristics (substrate type, sediment texture, and slope), and ecological associations.

## Results

### Modeling the species-environmental relationships

**Fig. 1:**
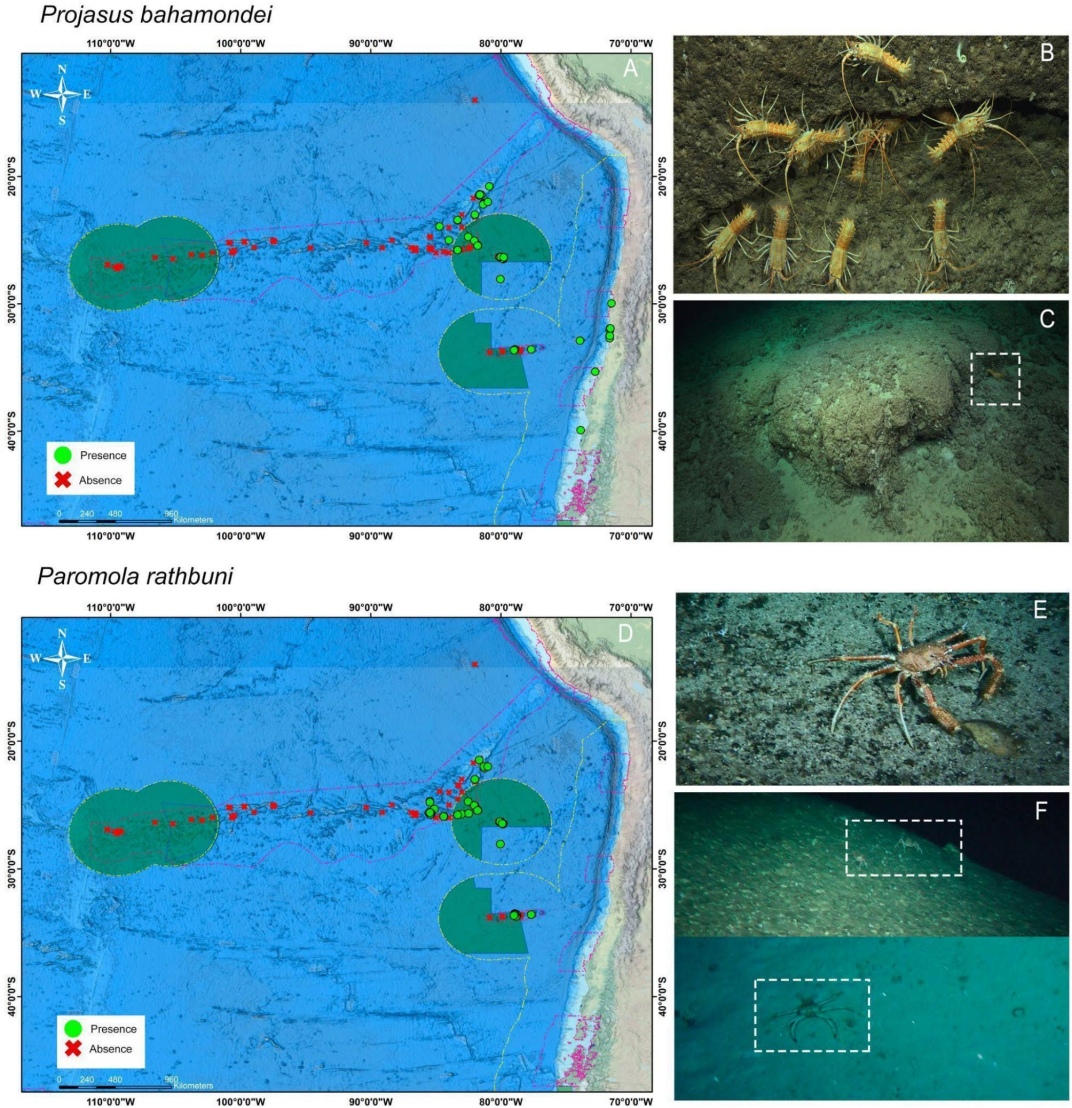
Distribution and presence of the target crustacean species, *Projasus bahamondei* (**A**–**C**) and *Paromola rathbuni* (**D**–**F**). Representative habitats where the species were observed in situ along the Salas and Gómez, Nazca and Juan Fernández ridges are shown. Green dots indicate presence, red crosses indicate absence. Map generated using ArcMap (version 10.8) and GEBCO and NCEI base maps (British Oceanographic Data Centre).

**Fig. 2:**
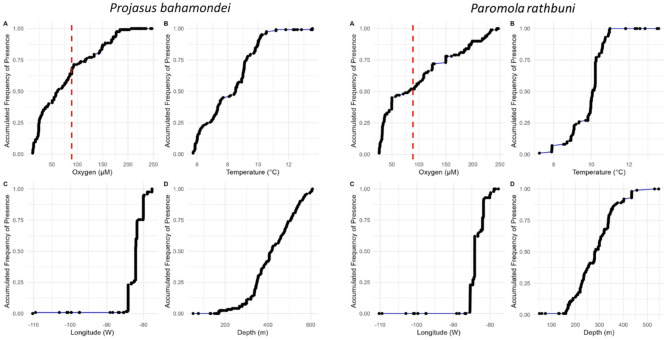
*Projasus bahamondei*: Accumulated frequency of *Projasus bahamondei*, as a function of dissolved oxygen (µM), temperature (°C), longitude (°W) and depth (m). *Paromola rathbuni*: Accumulated frequency of *Paromola rathbuni*, as a function of dissolved oxygen (µM), temperature (°C), longitude (°W) and depth (m). The red vertical line indicates the upper limit of the OMZ (2 ml L^–1^ = 89.2 µM).

**Fig. 3:**
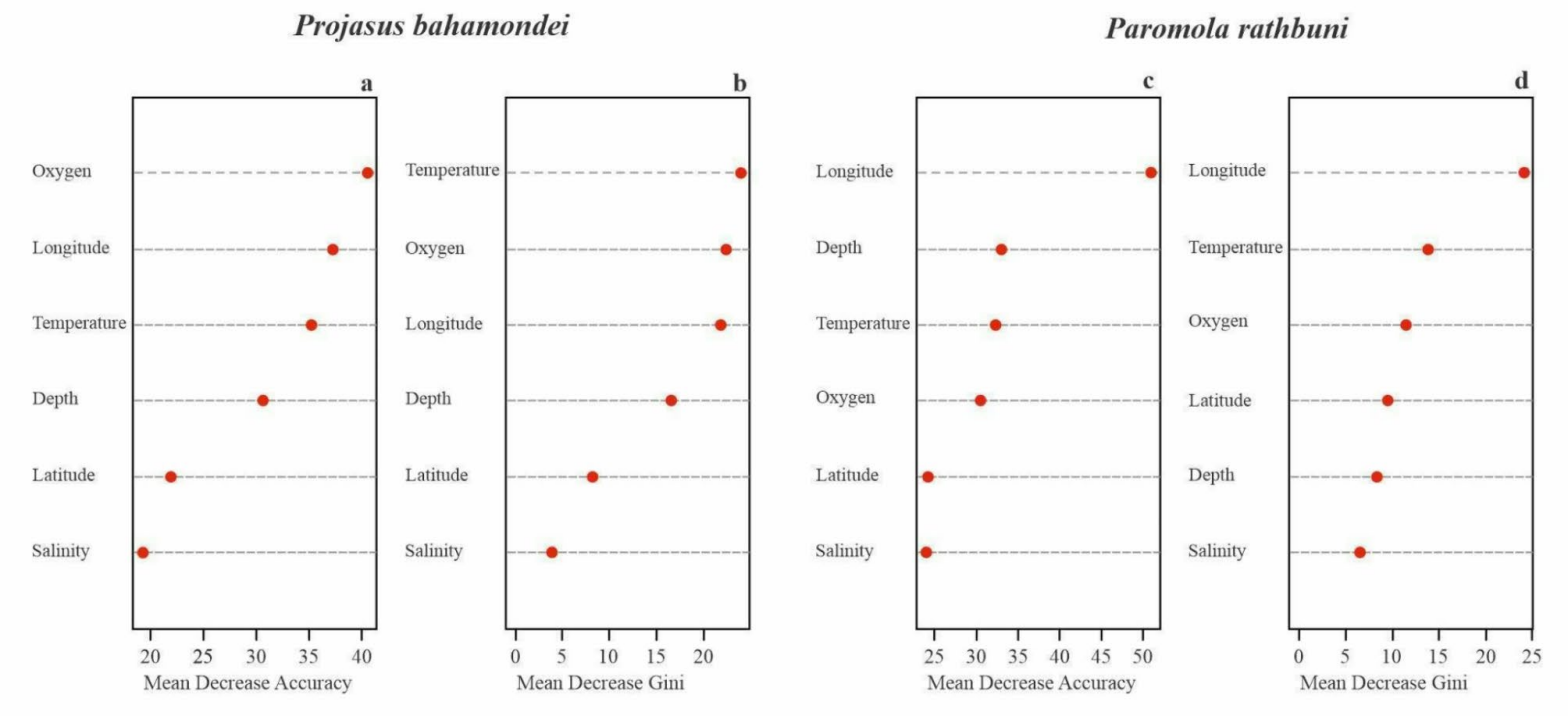
Model outputs for Random Forest classification method. The oceanographic variables (temperature, dissolved oxygen and salinity) and geographic variables (Longitude W, Latitude S, depth) are hierarchically ordered indicating the most important effects on the distribution of *Projasus bahamondei* (**a**,** b**) and *Paromola rathbuni* (**c**,** d**). Mean Decrease Accuracy is a metric that evaluates the importance of each variable. Mean Decrease Gini is a measure of the impurity of decision tree nodes; higher values indicate that the variable is important for reducing the impurity of the nodes.

The combinations of variables that allowed predicting the distribution of *P. bahamondei* and *P. rathbuni* through the S&G, NZ, and JF ridges (Figs. 1 and 2) differed between species. In general, the variables that most contribute to predicting the presence or absence of both species are dissolved oxygen, temperature, and longitude, but their importance within the ordering differs between species. The combined effects of geographic/geologic and physico-chemical variables explained 95% of the *P. bahamondei* distribution. The abiotic variables that contributed to high accuracy of the RF model (pseudo-R^2^ = 0.95) and low error rate (OOB = 4.93%) were dissolved oxygen, longitude (W), and temperature (mean decrease accuracy > 35), whereas, depth and latitude (S) had a secondary contribution (< 20 with mean decrease accuracy > 30) for the model (Fig. 3a,b).

The set of predictive variables that explained 93% the presence of the *P. rathbuni* and contributed to high accuracy of the model (pseudo-R^2^ = 0.93) and low error rate (OOB = 6.92%) were the combination of longitude (W) and depth (mean decrease accuracy > 35), whereas temperature and dissolved oxygen had a secondarily contribution (< 25 with mean decrease accuracy > 32) (Fig. 3c,d).

The best-fitting GLM (AIC = 84.83, R^2^ = 0.61) for the effects of physico-chemical variables on *P. bahamondei* explained 62% of the variance for presence data. The high predictive performance of the model (AUC = 0.97) indicates a high ability to discriminate between presence and absence data (Table 1). The presence of *P. bahamondei* was significantly (*p* < 0.001) affected by dissolved oxygen (low oxygen) and temperature (Table 1). Similarly, the best-fitting GLM for *P. rathbuni* (AIC = 152.8, R^2^ = 0.37) explained 37% of the variance for presence data, including a high predictive model performance (AUC = 0.83) (Table 2). The presence of *P. rathbuni* was significantly affected by low dissolved oxygen (*p* < 0.001) and low temperature (*p* < 0.001) (Table 2).

**Table 1:** Best-fitting GLMs used to evaluate the effects of physico-chemical variables (in situ temperature, dissolved oxygen and salinity) and geographic/geologic variables (Longitude W, latitude S, and depth) on the presence of *Projasus bahamondei* (Chilean Jagged lobster).

	Estimate	Std. error	z value	Pr(>|z|)	*p*
*Projasus bahamondei*					
glm (formula = Resp ~ O_2_ + Temp + 1, family = binomial(link = “logit”) AIC = 84.84; AUC = 0.97; R^2^ = 0.62					
Intercept	16.85	2.70	6.25	4.18e−10	***
O_2_	− 0.03	0.01	− 5.81	6.18e−09	***
Temp	− 1.39	0.26	− 5.29	1.25e−07	***
glm (formula = Resp ~ depth + Lat + Lon + 1, family = binomial(link = “logit”) AIC = 122.2; AUC = 0.95; R^2^ = 0.54					
Intercept	24.69962	8.05818	3.065	0.002176	**
Depth	0.01809	0.00276	6.557	5.5e−11	***
Lon	0.28995	0.08023	3.614	0.000302	***
Lat	0.22026	0.22026	0.22026	0.22026	+

Lon: longitude. O_2_: oxygen. Temp: temperature. Significance codes: not significant (+), *p* > 0.05 (.), *p* < 0.05(*), *p* < 0.01 (**), *p* < 0.001 (***). In bold the models with highest significance. AUC = Area under the curve, aic = akaike information criterion.

**Table 2:** Best-fitting GLMs used to evaluate the effects of physico-chemical variables (in situ temperature, dissolved oxygen and salinity) and geographic/geologic variables (Longitude W, latitude S, and depth) on the presence of *Paromola Rathbuni* (Juan Fernández crab).

	Estimate	Std. error	z value	Pr(>|z|)	*p*
*Paromola rathbuni*					
glm (formula = Resp ~ O_2_ + Temp + 1, family = binomial(link = “logit”) AIC = 152.8; AUC = 0.83, R^2^ = 0.33					
Intercept	10.3738	2.3001	4.510	6.48e−06	***
O_2_	− 0.013828	0.003196	− 4.327	1.51e−05	***
Temp	− 0.758694	0.224082	− 3.386	0.00071	***
glm (formula = Resp ~ depth + Lat + Lon + 1, family = binomial(link = “logit”) AIC = 155.6; AUC = 0.82, R^2^ = 0.32					
Intercept	27.986304	5.933177	4.717	2.39e−06	***
Depth	0.007072	0.002397	2.950	0.00317	**
Lat	0.288077	0.109102	2.640	0.00828	**
Lon	0.255481	0.052451	4.871	1.11e−06	***

Lon: longitude. O_2_: oxygen. Temp: temperature. Lat: latitude. Significance codes not significant (+), *p* > 0.05 (.), *p* < 0.05(*), *p* < 0.01 (**), *p* < 0.001 (***). In bold the models with highest significance. AUC = Area under the curve, aic = akaike information criterion

The best-fitting GLM (AIC = 122.4; R^2^ = 0.54) for the effects of geographic/geologic variables on *P. bahamondei* explained 54% of the variance for presence data, with high predictive model performance (AUC = 0.95). The presence of *P. bahamondei* was significantly (*p* < 0.001) affected by longitude and depth (Table 1). The best GLM (AIC = 155.6, R^2^ = 0.32) explained 32% of variance for *P. rathbuni* presence data, with high predictive performance (AUC = 0.82) (Table 2). The presence of *P. rathbuni* was significantly affected by longitude (*p* < 0.001), depth (*p* = 0.003), and latitude (*p* = 0.008) (Table 2).

### Habitat use and ecology in mobile deep-sea decapods

The target crustacean species were associated with two habitats types (Fig. 1): (i) Hard substrate, with sharp relief, including areas interspersed with patches of coarse sand and large rock formations, dominated by small sponges, lace corals (*Stylaster* sp.), and sea pens (*Scleroptilum* sp.). These habitats featured steep slopes with cracks providing shelter to numerous fish species (e.g., Synaphobranchidae, *Lotella fernandeziana* and *Scorpaena thompsoni*). (ii) Soft substrate, with little relief, composed of coarse sand and thanatocoenosis (foraminifera deposits and pteropod shells). This habitat was inhabited by Ceriantharidae and Hormathiidae anemones, sea pens (*Protoptilum* sp.), and various fish species (e.g., *Notopogon* sp., *Parapercis* sp., and *Squalus mitsukurii*).

The Chilean jagged lobster (*P. bahamondei*) was mostly associated with hard substrate and sharp relief (i.e., habitat) at depths of 150 to 608 m. Notably, during most dives, this species was observed forming aggregations of > 10 individuals on vertical walls or in caves (Fig. 1a–c). Conversely, the Juan Fernandez king crab (*P. rathbuni*) was found in both habitats, (i) hard (rocky) and (ii) soft (sandy) substrate, at depths of 50 to 400 m. This species was observed to be disaggregated as small groups of 2 to 3 individuals (Fig. 1d–f).

*Projasus bahamondei* was only found east of 84.2°W, with 50% of the records around 82°W and some records along the continental margin of Chile. This lobster was observed predominantly within the OMZ, where 75% of the records occurred in hypoxic conditions (< 2 mL L^− 1^ [< 89 µM]) at 150–500 m and within a narrow thermic range of 6–9 °C (Fig. 2). In contrast, *P. rathbuni* was distributed east of 85°W, with 50% of their records occurring at 84°W. A bout 50% of *P. rathbuni* records were registered under low oxygen conditions (< 2 mL L^− 1^), below 300 m depth, and at temperatures < 10 °C (Fig. 2). The presence of epibionts on the body of *P. bahamondei* and *P. rathbuni* was observed (see Fig. S1). Live cirripedians, including species such as *Poecilasma* spp., *Solidobalanus nascanus*, and *Verruca scrippsae*, were recorded attached to the antennae, cephalothorax, abdomen, and appendages of *P. bahamondei* and the carapace of *P. rathbuni*. Among the dominant epibionts inhabiting the studied crustaceans, *Poecilasma* spp. was particularly prevalent, with numbers exceeding 20 individuals on *P. bahamondei* cephalothorax. Interestingly, the presence of these cirripedians species was exclusively restricted to crustaceans collected on the NZ Ridge within the Nazca-Desventuradas Marine Park at the intersection of NZ and S&G and on the Desventuradas; individuals collected from the continental shelf of Chile and Juan Fernandez Archipelago were devoid of epibionts.

## Discussion

Our findings reveal that the target species are prevalent near the western extent of HCS at ~ 82–84 W^11^, located at the intersection of the S&G and NZ ridges, in areas with hypoxic conditions and colder temperatures, where higher concentrations of food should be available. RF and GLM models provided complementary evidence that indicates the presence of the OMZ on the NZ, S&G, and JF ridges is influencing the spatial and bathymetric distribution of both species, but more strongly for *P. bahamondei*. According to models, and that ~ 75% of observations occurred under hypoxic conditions, oxygen concentration is the most significant factor explaining the presence of *P. bahamondei* (R^2^= 0.61, GLM); the probability of finding *P. bahamondei* increases as dissolved oxygen concentration decreases. Thus, its longitudinal distribution in the NZ and S&G ridges does not extend beyond ~ 85°S, where the influence of the OMZ weakens and oxygen concentration exceeds 2 mL L^− 1^^8,9^. Its latitudinal and bathymetric distribution is also strongly influenced by the extent of the OMZ (Fig. 3). The association of *P. rathbuni* with the OMZ is not as strong since only 50% of the observations occurred under hypoxic conditions and temperatures below 10 °C. The low model variance (R^2^ = 0.37, GLM) suggests that other factors, in addition to the presence of the OMZ, may also shape the distribution of *P. rathbuni*.

The OMZ acts as an exclusion zone for many metazoan species lacking physiological and morphological adaptations to hypoxic environments. However, for OMZ residents, it provides a refuge, reduced predation, and a food source for detritivorous and generalist organisms^12,13^. In the S&G Ridge, the accumulation of organic material and water column productivity decrease from east to west^21,22^. Consequently, food availability declines sharply as the OMZ weakens, which could have significant impacts on populations of large organisms such as *P. bahamondei* and *P. rathbuni*.

In biogeographic terms, the discontinuity in the distribution of both decapods west of ~ 85°W (see Fig. 2) aligns with the presence of the biogeographic transition zone described by Parin et al.^11^ between 82°–84°W^11,23^, which generally separates Indo-Pacific benthic species to the west and temperate-origin fauna to the east^24^. West of the transition zone, the size of benthic megafauna tends to be smaller, suggesting that the size of potential prey would also be smaller and less abundant, whereas towards the east, prey would be larger and more available^11,25,26^.

Although both crustaceans are OMZ residents and are expected to have low metabolic rates, they are large-sized species in terms of volume, and require a higher total energy intake (food) compared to smaller decapods. Information on the dietary components of these decapods is limited with only one study on *P. bahamondei*. Báez et al.^26^ described the gastrointestinal contents of *P. bahamondei*, which included sediments and *Thioploca* sp. bacteria, foraminifera, gastropod mollusks, crustacean remains and eggs, shrimp, and fish from the family Gonostomatidae^26^. The presence of *Thioploca* sp. bacteria, a genus associated with hypoxic environments, suggests that *P. bahamondei* may feed in low-oxygen environments, such as those found in certain seamounts of the Nazca Ridge and at the S&G/NZ intersection^26,27^. The high incidence of foraminifera and crustacean remains suggests both detritivorous and carnivorous/scavenger feeding behaviors, indicating a generalist feeding habit, which is common among OMZ residents. Further insights on dietary habits of these species can be gained from dietary records for congeners such as *P. cuvieri*, a species from the Mediterranean Sea^28,29^. The gastrointestinal contents of *P. cuvieri* include chondrichthyan and teleost fish, pteropods, echinoids, decapod remains, and a small amount of foraminifera, along with detritus, as indicated by the high δ^15^N values observed^30–32^. Thus congeners also exhibit a generalist feeding habit, i.e., combination of detritivorous and carnivorous/scavenger feeding behaviors, common among OMZ residents.

### Mobile deep-sea decapods under different habitat characteristics

The benthic observations of the target species obtained from these expeditions allowed the description of the preferred habitat of each species and their ecological associations along the three ridges of the SEP. The lobster *P. bahamondei* was frequently associated with hard substrates and sharp relief, with a distribution across a wide depth range (150 to 608 m) and being more frequent and abundant on steep slopes (aggregations of > 10 individuals observed), similar to what was reported by Retamal and Arana^20^, Arana^19^, and Tapia-Guerra et al.^25^. In contrast, the Juan Fernández crab, *P. rathbuni*, was observed more frequently on sandy sediments (low roughness, flat or smoothed slope) and secondarily on rocky habitats (low roughness, vertical slope) within a narrower depth range (50 to 400 m). While both species were occasionally observed sharing habitat, each species is predominant in different habitats. Thus, slope, roughness, and sediment type may influence the presence and aggregation of these two deep-sea decapod species.

The presence and absence of epibionts such as the barnacle *Poecilasma* cf. *crassa* on the cephalothorax of both species may provide information about the connectivity between biogeographical zones of the SEP. This barnacle has been observed along the S&G Ridge on other brachyurans: *Chaceon chilensis*, *Hispidolambrus mirnovovi*, *Platepistoma balssii*, and *Cyrtomaia* spp. (Personal Observations), as well as on the target species of this study, indicating that it does not have a host preference. However, this epibiont is absent from the carapaces of *P. bahamondei* and *P. rathbuni* on the JF Ridge. *Poecilasma* cf. *crassa* has been described as a species with a circumtropical distribution and high dispersal potential^33^, so its absence on the JF Ridge (1,000 km south of the S&G/NZ) likely reflects a physiological limitation to the temperate conditions of the Juan Fernández Ecoregion rather than the dispersal potential of its planktonic phase or the absence of other hosts. Similarly, the absence of *P. bahamondei* and *P. rathbuni* west of 85°W is likely due to limitations in environmental tolerances (increased temperature and dissolved oxygen) rather than dispersal capacity in the larval or adult stage, as both species are present on the JF Ridge (1,000 km south of S&G/NZ). The family Palinuridae is considered to have high dispersal potential (> 6 months), which could explain the distribution of *P. bahamondei* on the Chilean continental shelf and out to the intersection of the NZ and S&G ridges and the JF Ridge. In contrast, the family Homolidae is also described as having high dispersal potential, but its planktonic phase lasts six times less than that of Palinuridae (30 days)^29,34^, which could explain the absence of *P. rathbuni* on the continental shelf of Chile.

Considering the results obtained and the projected climate change scenarios for this part of the SEP^8^, where alterations in temperature and oxygen conditions are expected, it is plausible that the geographic and bathymetric distribution patterns of *P. bahamondei* and *P. rathbuni* may change in the future. In particular, changes in the intensity and extent of the OMZ could affect the spatial distribution of these species along the three ridges studied.

## Conclusions

The distribution of both species is likely constrained by the contrasting oceanographic conditions of the biogeographic transition zone located between 82° and 85°W, where the confluence of subtropical water masses and temperate waters to the east occurs, including the weakening and disappearance of the OMZ influence. These conditions could limit the connectivity of larvae and adults to the west, possibly beyond their environmental tolerance ranges, preventing them from expanding westward despite their dispersal potential and the presence of preferred habitat. Given the mobility of adults, their generalist feeding habits, and dispersal potential, it is expected that under climate change conditions, including changes in oxygen and temperature, there could be expansions/contractions in the distribution of both species, depending on the direction of the projected change.

## Methods

### Study area

The S&G and NZ ridges are two adjacent chains of volcanic seamounts located in the southeastern Pacific, extending 3,000 km off the coast of Chile^4,7,9^. Rapa Nui (Easter Island) and Motu Motiro Hiva (Salas & Gómez Islet) in the west are the only summits that emerge from the S&G and NZ ridges^7^. To the east of the S&G and NZ intersection are the Desventuradas Islands (San Félix and Sand Ambrosio) and further south lies the JF ridge, which has ~ 15 seamounts and three emergent summits forming an archipelago composed of two islands (Robinson Crusoe, Alejandro Selkirk) and islets (Santa Clara)^5^.

The scientific information collected from these oceanic ridges comes mainly from Russian expeditions conducted between 1973 and 1987^11^. Additional information arises from campaigns led by Chile, including the CIMAR 5 (1999), CIMAR 6 (2000), CIMAR 22 (2016) cruises, as well as expeditions by National Geographic and Oceana (2013). More recently, information was collected from the EPIC campaign (2019) in collaboration with JAMSTEC (Japan)^2^ and the Schmidt Ocean Institute (SOI) expeditions aboard the R/V Falkor (too) in collaboration with ESMOI (Chile) and The University of Texas Rio Grande Valley (USA). These expeditions surveyed the three ridges at depths ranging from 150 to 4,500 m, with greater spatial distribution that the early expeditions, which were preliminary on the high sea (Fig. S2).

### Oceanographic conditions, circulation and water masses

The S&G, NZ, and JF ridges are located on the eastern edge of the SEP anticyclonic gyre, which is formed by the South Pacific Current to the east, the South Equatorial Current to the west and the Humboldt Current to the north^10^. Towards the east, the oceanographic conditions are highly influenced by the HCS, evidenced by low temperature, high nutrients concentration, and low dissolved oxygen concentration at the sub-surface level^8,10,35^. Towards the west, within the Easter Island ecoregion^36^, the waters extending from Motu Motiro Hiva (Salas & Gómez islet) to Rapa Nui are characterized by increasing temperature and dissolved oxygen concentration and decreasing nutrient concentration^8,9^. Oligotrophic conditions promote high water clarity, allowing the passage of light at greater depths than in other areas, resulting in the presence of mesophotic communities (e.g. crustose coralline algae) at depths of > 300 m on the S&G ridge^1,4,8^.

Over the S&G ridge (~ 25–26°S) the confluence of several water masses occurs along two axes: bathymetric and longitudinal. In the surface layer (between 0 and 200 m), the Subtropical Water (STW) is situated to the west, while the Sub-Antarctic Water (SAW) is dominating to the east^8^. Below 200 m the presence of the Equatorial Subsurface Water (ESSW) characterized by low levels of dissolved oxygen (O_2_ < 44.6 µmol/L), high salinity (34.9 PSU) and high nutrients concentrations^8,10^, is associated with the core of the OMZ. To the east the OMZ reaches values less than 4.5 µmol/L at 200 m but increases its depth towards the west, reaching 380 m, before disappearing west of 82°W^8,9,35^.

### Deep-sea megafauna under study

In general, species of family Palinuridae possess a long larval phase comprising from 11 to 17 planktonic larval stages (phyllosoma and puerulus), with high dispersal potential due to an estimated planktonic duration of 6 to 11 months^37^ during which time they can travel large distances with high dispersal potential^38^. *Projasus bahamondei* is distributed along the NZ ridge and the eastern portion of the S&G Ridge, mainly east of ~ 84°W^11^, where it has been highly commercially exploited by industrial vessels operating in international waters off the coast of Peru, mainly between 20°00′ S–24°00′ S and 79°50′ W–84°50′ W^19^. It has also been found in the JF Ridge^19^ and Desventuradas islands (286–400 m). The presence of *P. bahamondei* is registered occasionally in the continental margin of Chile^20^ (Retamal and Arana, 2000) off Iquique (170–550 m), central coast of Valparaíso^39^, and off Huasco (28°28′ S) and Constitución (35°20′ S)^19,20,40^. On the Chilean continental shelf, *P. bahamondei* is incidentally caught, mainly in trawl fisheries targeting *Heterocarpus reedi* (nylon shrimp), *Grimothea johni* (yellow squat lobster) and *Grimothea monodon* (red squat lobster)^41^. This lobster species has been described in both sandy and rocky bottom habitats, typically on the summits of seamounts^11,19,25^.

*Paromola rathbuni* is endemic to the ridges of the SEP, being described from the JF Ridge^42,43^ to the Desventuradas Islands, with a bathymetric distribution between 100 and 300 m and in association with muddy or sandy bottoms^19,25,43,44^. It is incidentally captured as bycatch in the artisanal fisheries targeting *Chaceon chilensis* in the JF Ridge^45,46^.

### Scientific cruises: operational characteristics

The operational information of the onboard equipment, sampling procedure, and the data collected during the EPIC 2019, CIMAR 22, FKt240108, FKt240224, FKt240708 cruises, as well the analysis of video records and samples processing on laboratory, are described in the supplementary material. Geographical and bathymetric presence, ecological observations, and habitat characteristics of the studied decapod crustaceans were obtained from the observation of benthic videos recorded during each cruise (Fig. 2S; see details in the supplementary material). The habitat was characterized by substrate type (e.g., rock, silty sediments, mixed substrate, coarse sand), “modifier” elements (e.g., biological communities, sedimentation, and bioturbation), and associated fauna following Greene et al.^47^ and Tapia-Guerra et al.^25^.

### Sampling and data collection

#### Oceanographic data

The measurements of temperature (°C), salinity (PSU), and dissolved oxygen (µmol/L) were collected in situ during four of the five cruises (EPIC 2019, CIMAR 22, FKt240108, FKt240224, and FKt240708). For the CIMAR 22 cruise and due to logistical constraints (non-calibrated CTD), the abiotic variables (temperature, salinity, and dissolved oxygen) were obtained from Mecho et al.^9^, whose processed data from CSIRO Atlas of Regional Seas Climatology 2009 database. The metadata detailing the equipment deployed during the five cruises are provided in Table 3 and the oceanographic variables measured in situ are described in Table 4. Additional details on the oceanographic variables are available in Table S1. The metadata characterizing the ecology and habitat features associated with the target species (*P. bahamondei* and *P. rathbuni*) are summarized in Table 5 and further detailed in Table S2.

**Table 3:** Metadata to describe operational information of the equipment displayed during the EPIC 2019, CIMAR 22, FKt240108, FKt240224, FKt240708 cruises.

Year	Cruise	Agassiz trawl characteristics	Submarine videos records	Biological samples	Sets with Trawl (n°)	Dives with DT (*n*°)	Dives with ROV (*n*°)
Operational information							
2016	CIMAR 22	Opening: 1.5m × 0.5 m	ROV Commander MK2	Yes	15	NC	11
		Mesh size: 12 mm					
		Total sweep time: Yes					
2019	EPIC 2019	Opening: 2 m × 0 × 5 m	DT	Yes	6	9	NC
		Mesh size: 12 mm					
		Total sweep time: Yes					
2024	FKt240108	NC	ROV SuBastian	Yes	NC	NC	20
2024	FKt240224	NC	ROV SuBastian	Yes	NC	NC	26
2024	FKt240708	NC	ROV SuBastian	Yes	NC	NC	13

DT, deep tow; ROV, remotely operated vehicle; NC, not corresponding.

**Table 4:** Metadata describing geographic and physicochemical variables measured in situ during five high sea cruises (CIMAR 22, EPIC 2019, FKt240108, FKt240224, FKt240708).

Year	Cruise	Lat (S)		Lon (W)		Ts (°C)		Tb (°C)		Salinity (PSU)		DO (µM)		Depth (m)	
		Min	Max	Min	Max	Min	Max	Min	Max	Min	Max	Min	Max	Min	Max
2016	CIMAR 22	24.7	33.8	77.7	83.3	16.6	19.4	8.6	10	34	34.7	56.7	244.6	50	370
2019	EPIC 19	25.4	26.5	79.8	102.9	20.8	25	9.1	13.5	34.3	34.9	83.6	215.1	208	547
2024	FKt240108	24.7	33.7	77.6	86.6	20	12.5	2.1	13	34.3	34.7	25	325	178	2008
2024	FKt240224	21.3	27.2	81.2	110.2	17.5	25	5	17.5	34.3	35.7	150	360	200	1381
2024	FKt240708	14.2	25.7	81.2	85.3	17.5	18	4.0	12	34.4	34.6	25	300	230	4500

The ranges for each variable are shown. Ts, surface temperature; Tb, bottom temperature; DO, dissolved oxygen; Min, minimum; Max maximum.

**Table 5:** Ecological information for *Projasus bahamondei* and *Paromola rathbuni*, as well as habitat characteristics of the systems where the species were observed during the cruises (EPIC 2019, CIMAR 22, FKt240108, FKt240224, FKt240708).

Cruise/Species	Latitude (S)		Longitude (W)		Depth (m)		Presence or Absence in Subsystem		Slope			Sediment type		Sediment roughness		
	Min	Max	Min	Max	Min	Max	Island	Seamount	Flat	Sloping	Vertical	Rock	Sand	High	Medium	Low
CIMAR22	24.7	26.4	79.8	83.3	200	350										
* P. rathbuni*	24.7	25.7	80.0	82.3	150	340	P	P	P	P	A	P	P	P	P	P
Both species	24.7	26.2	79.8	82.5	280	340	P	P	P	P	P	P	A	P	P	A
Both species	24.7	24.7	82.5	82.5	280	280	A	P	P	P	P	P	A	P	A	A
EPIC19	25.4	26.5	79.8	81.8	150	1.050										
* P. rathbuni*	25.4	26.4	79.8	81.7	229	371	P	P	P	P	A	P	P	P	P	P
* P. bahamondei*	25.4	26.4	79.8	81.7	350	684	P	P	P	P	P	P	A	P	P	A
Both species	–	–	–	–	–	–	A	A	A	A	A	A	A	A	A	A
FKt240108	21.4	33.5	77.6	82.7	178	2,008										
* P. rathbuni*	25.0	25.4	81.7	82.0	222	456	–	P	P	P	A	P	P	P	P	P
* P. bahamondei*	25.0	33.5	77.6	82.0	369	608	–	P	P	P	P	P	A	P	P	A
Both species	–	–	–	–	–	–	A	A	A	A	A	A	A	A	A	A
FKt240224	26.9	25.1	90.5	101.2	100	1,387										
* P. rathbuni*	–	–	–	–	–	–	A	A	A	A	A	A	A	A	A	A
* P. bahamondei*	–	–	–	–	–	–	A	A	A	A	A	A	A	A	A	A
Both species	–	–	–	–	–	–	A	A	A	A	A	A	A	A	A	A
FKt240708	14.1	25.8	81.2	84.7	274	4,500										
* P. rathbuni*	22.1	25.4	81.2	84.1	220	396	–	P	P	P	P	P	P	P	P	P
* P. bahamondei*	22.1	25.4	81.2	84.1	250	564	–	P	P	P	P	P	A	P	P	A
Both species	22.1	25.4	81.2	84.2	290	337	–	P	P	P	A	P	A	P	P	A

Min, minimum; Max, maximum; P, presence; A, absence. Dash (–) indicates no data.

#### Biological and ecology data

The benthic specimens collected during the five cruises were preliminarily sorted, counted, photographed, labeled, and preserved in 95% ethanol aboard the vessels. Subsequently, the samples were transferred to the Sala de Colecciones Biológicas at Universidad Católica del Norte (SCBUCN), where they were taxonomically identified to the lowest possible taxon, asigned an identification code, and cataloged. The identification of the target species was confirmed using the description guides for *P. rathbuni*^20,24,44,48^ and *P. bahamondei*^19,24,48,49^.

A bibliographic review of *P. bahamondei* and *P. rathbuni* was conducted to complement data on presence, distribution, and ecology. The review included data from previous expeditions, artisanal fishing data, online databases, and scientific articles (Fig. S3). For each record, the collected data included year, station, latitude, longitude, depth, species, subsystem type, and bibliographic source (see Tables S1, S3).

### Statistical modeling

Data modeling was performed using RStudio software, version 4.3.3^50^. The Random Forest (RF) method (one model per species) was applied for classification and regression^51,52^ to estimate whether ensembles of physicochemical variables (bottom temperature, dissolved oxygen, and salinity) and geographic/geologic variables (longitude [W], latitude [S], and depth) significantly influence the distribution of *P. bahamondei* and *P. rathbuni*. The RF allows the most important abiotic (predictive) variables to be hierarchically ordered to explain the distribution of each species separately. Model accuracy (pseudo-R^2^) was evaluated, and the model error rate (out-of-bag error, OOB), which represents the prediction error that occurs when RF takes into account a set of variables that were not selected, was calculated account^51,52^. The number of variables selected in each partition of each RF tree was two, the default value. The mean decrease in accuracy per variable was calculated, applying random permutations, to estimate the relative importance of the predictive variables. The mean decrease in the Gini coefficient was calculated to measure how each variable contributes to the homogeneity of the nodes and leaves in the resulting RF models. If the variable is useful, it tends to split mixed labeled nodes into pure single class nodes. Splitting by a permuted variable tends neither to increase nor decrease node purities. The RF analysis was run using the “randomForest” package version 4.6.12^52^.

Generalized Linear Models (GLM) were conducted to model the functional relationship between the independent variables (environmental and geographic) and the dependent variable (presence/absence), as well as to quantify how each variable directly or indirectly affects the probability of presence/absence for each species. Two GLMs were run; one including physico-chemical variables and the other containing geographic/geologic variables as independent (explanatory) variables. Prior to GLM runs, a multiple correlation analysis between abiotic variables and a multicollinearity between independent variables was evaluated using the Variance Inflation Factor (VIF; Table S4), a useful measure to detect multicollinearity between variables within a correlation matrix (Library Car, version 3.1-2)^53^. Highly correlated abiotic variables (*r* > 0.5) and with high collinearity (VIF > 5) were excluded as simultaneous explanatory variables to build the GLMs. The correlation between abiotic variables was not significant *r* < 0.5, except for salinity and temperature correlation at both data sets (*P. rathbuni*: *r* = 0.58; *P. bahamondei*: *r* = 0.53; Table S4). In turn, low collinearity between abiotic variables was detected within the correlation matrix for both data sets (*P. rathbuni*: VIF < 1.25; *P. bahamondei*: VIF < 2.33; Table S4). The frequentist statistical approach was used to fit the GLM method^54^. The MuMIn package (version 1.48.4^55^) was used to select the best-performing model from a set of candidate models. The GLMs were built by applying the binomial family with a logit link function. The Akaike Information Criterion (AIC) was used to identify the best model. The goodness of fit for the best model was evaluated to provide an indication of the proportion of variance explained by the model. The predictive model performance was assessed using the Area Under the Curve (AUC), which is a measure of the model’s ability to discriminate between presence and absence observations, of the Receiver Operating Characteristic (ROC) curve with the pROC package (version 1.18.5^56^).

To create graphs and visual results, the “ggplot2” data package version 3.4.3^57^ was used.

## Electronic supplementary material

Below is the link to the electronic supplementary material.

Supplementary Material 1

Supplementary Material 2

## Data Availability

All data generated or analysed during this study are included in the manuscript and its supplementary information files.
