# Supplementary material for: Spatial ecology of two emblematic deep-sea crustaceans in the Salas y Gómez, Nazca and Juan Fernández ridges Southeast Pacific
Source: Sci Rep. 2025 Apr 25;15:14538. doi: 10.1038/s41598-025-98820-4 (PMC12032079; doi:10.1038/s41598-025-98820-4)
Supplement: Supplementary file 2 — Supplementary Material 2 [file 41598_2025_98820_MOESM2_ESM.docx]

**SUPPLEMENTAL MATERIAL**

**Spatial ecology of two emblematic crustacean species in deep-sea ecosystems at Salas & Gómez, Nazca and Juan Fernández ridges (Southeast Pacific)**

Maximiliano Fernández-Zúñiga, Rodolfo Vögler, María de los Ángeles Gallardo^*^, Jan M. Tapia-Guerra, Javier Sellanes


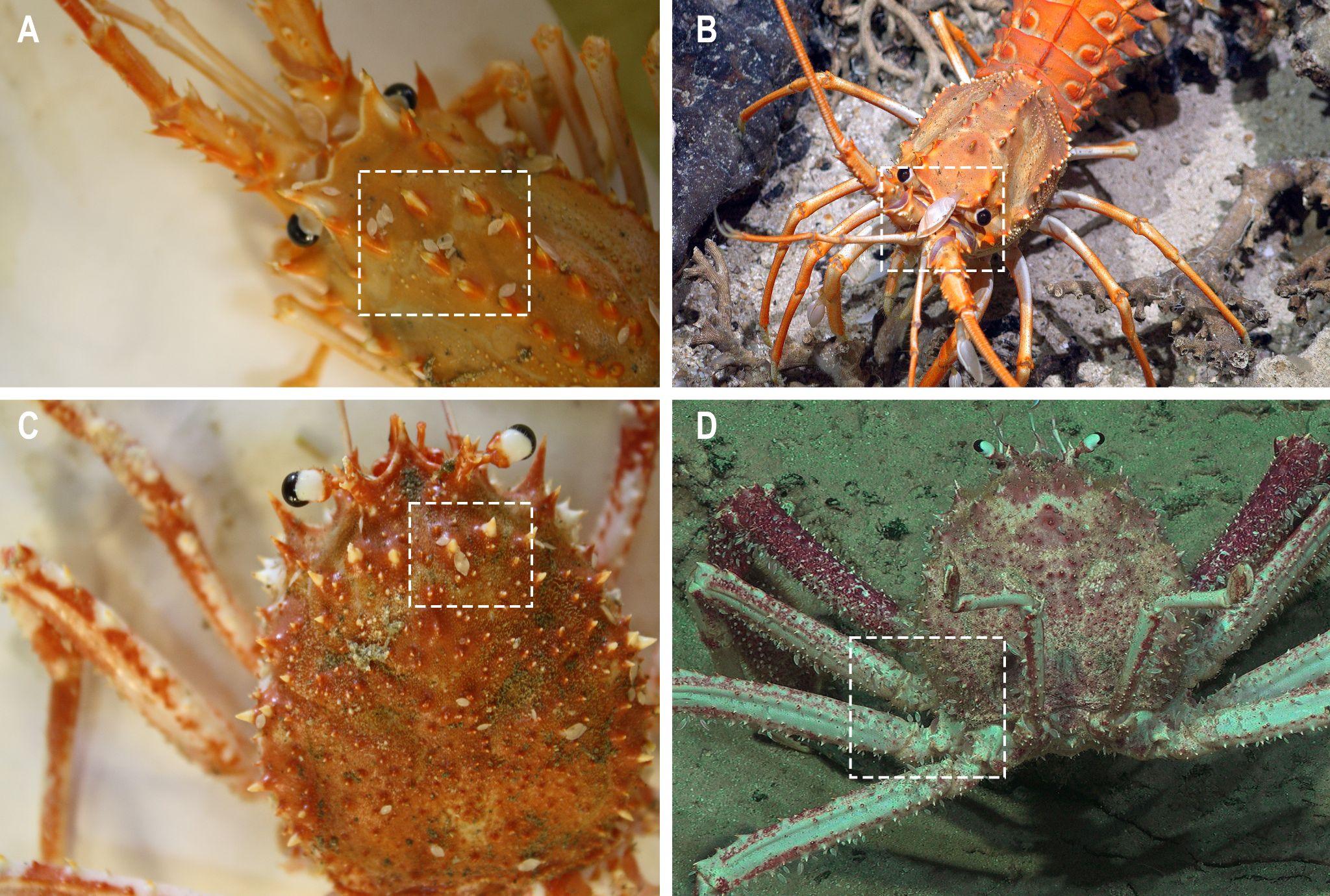


**Figure S1.** Living epibionts (white dotted lines) detected on the exoskeleton of *Projasus bahamondei* (A-B) and *Paromola rathbuni* (C-D). The images were obtained through direct observations of ROV.

**
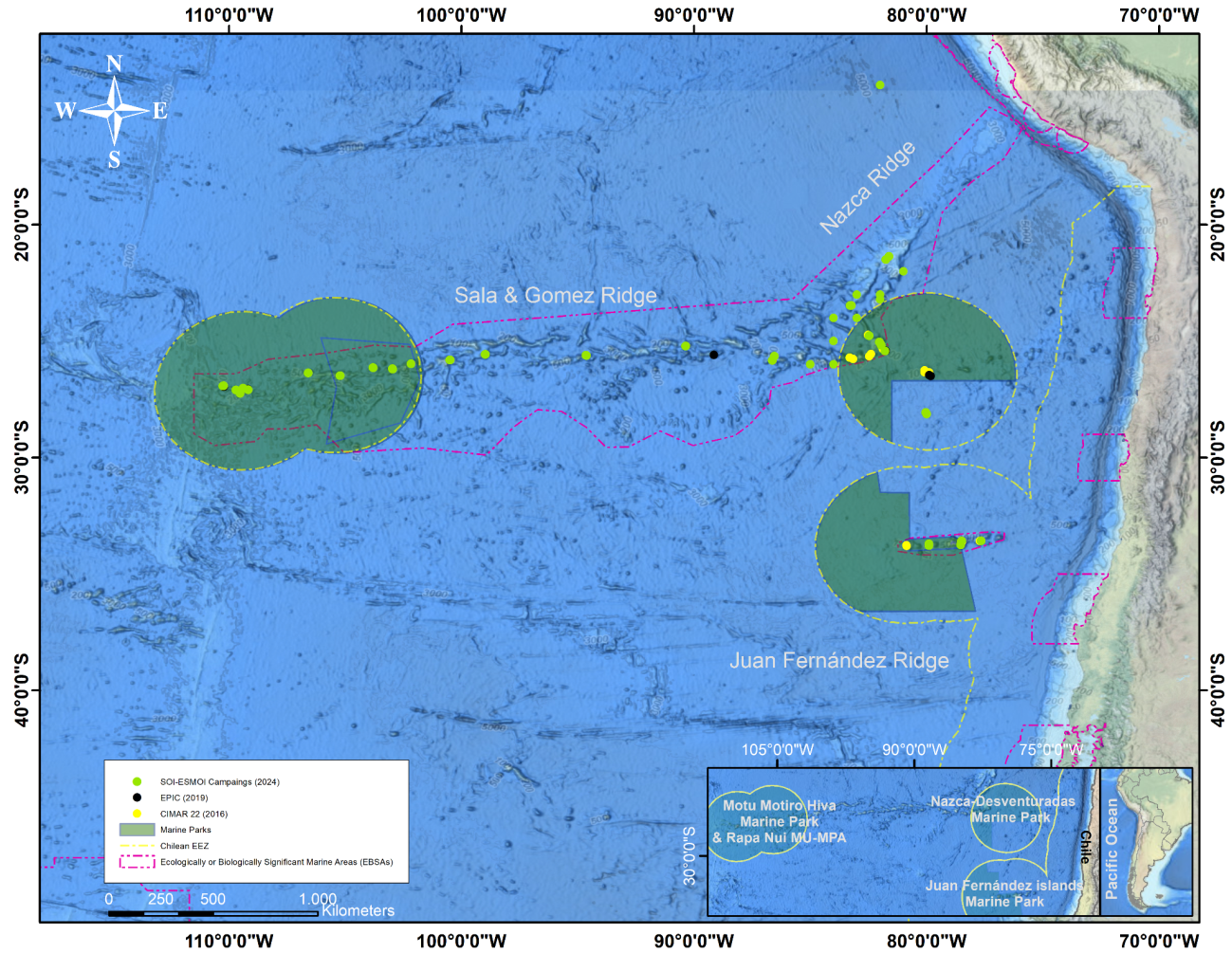
**

**Figure S2.** Sampling stations for the scientific cruises EPIC 2019 (black dots), CIMAR 22 (yellow dots), SOI-ESMOI 2024 (FKt240108, FKt240224 and FKt240708; green dots), carried out in the region of the Nazca, Salas & Gómez and Juan Fernández ridges. The marine parks (green area) of Motu Motiro Hiva, Nazca-Desventuradas and Mar de Juan Fernández, as well as the Exclusive Economic Zone of Chile (yellow dotted lines), and Ecologically or Biologically Significant Marine areas (purple dotted lines) are indicated. Map generated using ArcMap (version 10.8) and GEBCO and NCEI base maps (British Oceanographic Data Centre). Note that when multiple dives were recorded during a survey, points overlap.

**
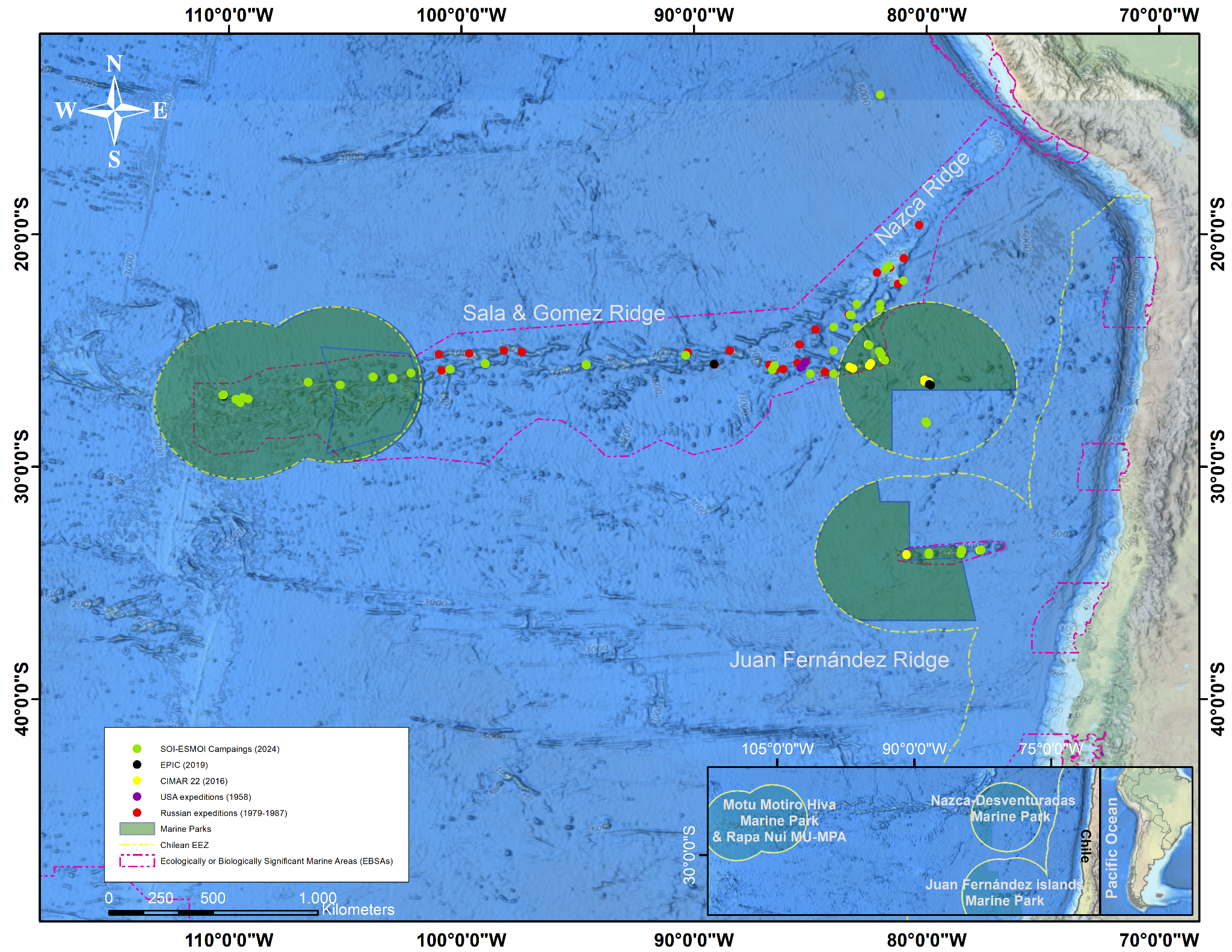
**

**Figure S3.** Spatial distribution of all records obtained during scientific expeditions carried out in the region of Nazca, Salas & Gómez and Juan Fernández ridges, Southeast Pacific. The research cruises include Russian expeditions (red), USA expeditions (purple), CIMAR 22 (yellow), EPIC 2019 (black), SOI-ESMOI 2024 (FKt240108, FKt240224 and FKt240708; green dots). The Motu Motiro Hiva, Nazca-Desventuradas and Mar de Juan Fernández Marine Parks (green area), as well as the Exclusive Economic Zone of Chile (yellow dotted lines) are indicated. Map generated using ArcMap (version 10.8) and GEBCO and NCEI base maps (British Oceanographic Data Centre, 2008; NCEI, 2023). Note that when multiple dives were recorded during a survey, points overlap.

**Table S1.** *In situ* measurements of temperature (°C), salinity (PSU) and dissolved oxygen (µM). This set of abiotic variables were obtained during the most recent high sea expeditions: EPIC 2019, CIMAR 22, SOI-ESMOI (FKt240108-Fkt240224-FKt240708).

*See excel file Table S1*

**Table S2.** Ecological information (distribution; abundance; geographic position: latitude, longitude) collected for *Projasus bahamondei* and *Paromola rathbuni* as well the habitat characteristics (slope; sediment: type, texture) by species which were observed *in situ* during different scientific campaigns (EPIC 2019, CIMAR 22, FKt240108 and FKt240224).

| **Distribution of *Paramola rathbuni* and *Projasus bahamondei* and characterization of habitats** | | | | | | | | | |
| --- | --- | --- | --- | --- | --- | --- | --- | --- | --- |
| **Date** | **Cruise** | **Taxa** | **Lat (S)** | **Lon (W)** | **Depth (m)** | **System** | **Slope** | **Sediment type** | **Sediment texture** |
| 2016 | CIMAR22 | *P. rathbuni* | -24,7 | -82,5 | 289 | seamount | flat | rocks | high rugosity |
| 2016 | CIMAR22 | *P. bahamondei* | -24,7 | -82,5 | 289 | seamount | flat | rocks | high rugosity |
| 2016 | CIMAR22 | *P. rathbuni* | -24,7 | -82,5 | 289 | seamount | flat | rocks | high rugosity |
| 2016 | CIMAR22 | *P. bahamondei* | -24,7 | -82,5 | 289 | seamount | sloping | rocks | high rugosity |
| 2016 | CIMAR22 | *P. bahamondei* & *P. rathbuni* | -24,7 | -82,5 | 289 | seamount | flat | rocks | high rugosity |
| 2016 | CIMAR22 | *P. bahamondei* | -24,7 | -82,5 | 289 | seamount | sloping | rocks | high rugosity |
| 2016 | CIMAR22 | *P. bahamondei* & *P. rathbuni* | -24,7 | -82,5 | 289 | seamount | sloping | rocks | high rugosity |
| 2016 | CIMAR22 | *P. rathbuni* | -24,7 | -82,5 | 289 | seamount | sloping | rocks | high rugosity |
| 2016 | CIMAR22 | *P. rathbuni* | -24,7 | -82,5 | 289 | seamount | sloping | rocks | high rugosity |
| 2016 | CIMAR22 | *P. bahamondei* | -24,7 | -82,5 | 289 | seamount | sloping | rocks | high rugosity |
| 2016 | CIMAR22 | *P. bahamondei* | -24,7 | -82,5 | 289 | seamount | sloping | rocks | high rugosity |
| 2016 | CIMAR22 | *P. rathbuni* | -24,7 | -82,5 | 289 | seamount | sloping | rocks | high rugosity |
| 2016 | CIMAR22 | *P. bahamondei* | -24,7 | -82,5 | 289 | seamount | vertical | rocks | high rugosity |
| 2016 | CIMAR22 | *P. rathbuni* | -24,7 | -82,5 | 289 | seamount | sloping | rocks | high rugosity |
| 2016 | CIMAR22 | *P. rathbuni* | -24,7 | -82,5 | 289 | seamount | flat | rocks | high rugosity |
| 2016 | CIMAR22 | *P. bahamondei* | -24,7 | -82,5 | 289 | seamount | flat | rocks | high rugosity |
| 2016 | CIMAR22 | *P. bahamondei* & *P. rathbuni* | -24,7 | -82,5 | 289 | seamount | flat | rocks | high rugosity |
| 2016 | CIMAR22 | *P. bahamondei* | -24,7 | -82,5 | 289 | seamount | vertical | rocks | high rugosity |
| 2016 | CIMAR22 | *P. bahamondei* | -24,7 | -82,5 | 289 | seamount | vertical | rocks | high rugosity |
| 2016 | CIMAR22 | *P. bahamondei* | -24,7 | -82,5 | 289 | seamount | flat | rocks | high rugosity |
| 2016 | CIMAR22 | *P. bahamondei* | -24,7 | -82,5 | 289 | seamount | flat | rocks | high rugosity |
| 2016 | CIMAR22 | *P. bahamondei* | -24,7 | -82,5 | 289 | seamount | flat | rocks | high rugosity |
| 2016 | CIMAR22 | *P. bahamondei* & *P. rathbuni* | -24,7 | -82,5 | 289 | seamount | flat | rocks | high rugosity |
| 2016 | CIMAR22 | *P. bahamondei* & *P. rathbuni* | -24,7 | -82,5 | 289 | seamount | flat | rocks | high rugosity |
| 2016 | CIMAR22 | *P. bahamondei* & *P. rathbuni* | -24,7 | -82,5 | 289 | seamount | flat | rocks | high rugosity |
| 2016 | CIMAR22 | *P. rathbuni* | -24,7 | -82,5 | 286 | seamount | sloping | rocks | high rugosity |
| 2016 | CIMAR22 | *P. rathbuni* | -24,7 | -82,5 | 286 | seamount | sloping | rocks | high rugosity |
| 2016 | CIMAR22 | *P. bahamondei* | -24,7 | -82,5 | 286 | seamount | sloping | rocks | high rugosity |
| 2016 | CIMAR22 | *P. rathbuni* | -24,7 | -82,5 | 286 | seamount | sloping | rocks | high rugosity |
| 2016 | CIMAR22 | *P. bahamondei* | -24,7 | -82,5 | 286 | seamount | flat | rocks | high rugosity |
| 2016 | CIMAR22 | *P. rathbuni* | -24,7 | -82,5 | 286 | seamount | flat | rocks | high rugosity |
| 2016 | CIMAR22 | *P. bahamondei* | -24,7 | -82,5 | 286 | seamount | sloping | rocks | high rugosity |
| 2016 | CIMAR22 | *P. rathbuni* | -24,7 | -82,5 | 286 | seamount | sloping | rocks | high rugosity |
| 2016 | CIMAR22 | *P. rathbuni* | -24,7 | -82,5 | 286 | seamount | sloping | rocks | high rugosity |
| 2016 | CIMAR22 | *P. rathbuni* | -24,7 | -82,5 | 286 | seamount | flat | rocks | high rugosity |
| 2016 | CIMAR22 | *P. rathbuni* | -24,7 | -82,5 | 286 | seamount | flat | rocks | high rugosity |
| 2016 | CIMAR22 | *P. rathbuni* | -24,7 | -82,5 | 286 | seamount | flat | rocks | high rugosity |
| 2016 | CIMAR22 | *P. bahamondei* & *P. rathbuni* | -24,7 | -82,5 | 286 | seamount | vertical | rocks | high rugosity |
| 2016 | CIMAR22 | *P. rathbuni* | -24,7 | -82,5 | 286 | seamount | vertical | rocks | high rugosity |
| 2016 | CIMAR22 | *P. bahamondei* & *P. rathbuni* | -24,7 | -82,5 | 286 | seamount | sloping | rocks | high rugosity |
| 2016 | CIMAR22 | *P. rathbuni* | -24,7 | -82,5 | 286 | seamount | flat | rocks | high rugosity |
| 2016 | CIMAR22 | *P. bahamondei* | -24,7 | -82,5 | 286 | seamount | flat | rocks | high rugosity |
| 2016 | CIMAR22 | *P. rathbuni* | -24,7 | -82,5 | 286 | seamount | flat | sand | medium rugosity |
| 2016 | CIMAR22 | *P. rathbuni* | -24,7 | -82,5 | 286 | seamount | sloping | rocks | high rugosity |
| 2016 | CIMAR22 | *P. rathbuni* | -24,7 | -82,5 | 286 | seamount | flat | rocks | high rugosity |
| 2016 | CIMAR22 | *P. bahamondei* | -24,7 | -82,5 | 286 | seamount | flat | rocks | high rugosity |
| 2016 | CIMAR22 | *P. bahamondei* | -24,7 | -82,5 | 286 | seamount | flat | rocks | high rugosity |
| 2016 | CIMAR22 | *P. bahamondei* & *P. rathbuni* | -24,7 | -82,5 | 286 | seamount | flat | rocks | high rugosity |
| 2016 | CIMAR22 | *P. bahamondei* | -24,7 | -82,5 | 286 | seamount | flat | rocks | high rugosity |
| 2016 | CIMAR22 | *P. rathbuni* | -24,7 | -82,5 | 286 | seamount | flat | rocks | high rugosity |
| 2016 | CIMAR22 | *P. bahamondei* | -24,7 | -82,5 | 280 | seamount | sloping | rocks | high rugosity |
| 2016 | CIMAR22 | *P. bahamondei* | -24,7 | -82,5 | 280 | seamount | sloping | rocks | high rugosity |
| 2016 | CIMAR22 | *P. bahamondei* & *P. rathbuni* | -24,7 | -82,5 | 280 | seamount | sloping | rocks | high rugosity |
| 2016 | CIMAR22 | *P. bahamondei* | -24,7 | -82,5 | 280 | seamount | sloping | rocks | high rugosity |
| 2016 | CIMAR22 | *P. bahamondei* | -24,7 | -82,5 | 280 | seamount | sloping | rocks | high rugosity |
| 2016 | CIMAR22 | *P. bahamondei* | -24,7 | -82,5 | 280 | seamount | flat | rocks | high rugosity |
| 2016 | CIMAR22 | *P. bahamondei* & *P. rathbuni* | -24,7 | -82,5 | 280 | seamount | flat | rocks | high rugosity |
| 2016 | CIMAR22 | *P. bahamondei* | -24,7 | -82,5 | 280 | seamount | flat | rocks | high rugosity |
| 2016 | CIMAR22 | *P. rathbuni* | -24,7 | -82,5 | 280 | seamount | flat | rocks | high rugosity |
| 2016 | CIMAR22 | *P. bahamondei* | -24,7 | -82,5 | 280 | seamount | flat | rocks | high rugosity |
| 2016 | CIMAR22 | *P. bahamondei* | -24,7 | -82,5 | 280 | seamount | flat | rocks | high rugosity |
| 2016 | CIMAR22 | *P. bahamondei* | -24,7 | -82,5 | 280 | seamount | flat | rocks | high rugosity |
| 2016 | CIMAR22 | *P. bahamondei* | -24,7 | -82,5 | 280 | seamount | sloping | rocks | high rugosity |
| 2016 | CIMAR22 | *P. bahamondei* | -24,7 | -82,5 | 280 | seamount | sloping | rocks | high rugosity |
| 2016 | CIMAR22 | *P. rathbuni* | -24,7 | -82,5 | 280 | seamount | sloping | rocks | high rugosity |
| 2016 | CIMAR22 | *P. bahamondei* | -24,7 | -82,5 | 280 | seamount | sloping | rocks | high rugosity |
| 2016 | CIMAR22 | *P. bahamondei* | -24,7 | -82,5 | 280 | seamount | sloping | rocks | high rugosity |
| 2016 | CIMAR22 | *P. bahamondei* & *P. rathbuni* | -24,7 | -82,5 | 280 | seamount | sloping | rocks | high rugosity |
| 2016 | CIMAR22 | *P. bahamondei* & *P. rathbuni* | -24,7 | -82,5 | 280 | seamount | sloping | rocks | high rugosity |
| 2016 | CIMAR22 | *P. bahamondei* | -24,7 | -82,5 | 280 | seamount | sloping | rocks | high rugosity |
| 2016 | CIMAR22 | *P. bahamondei* & *P. rathbuni* | -24,7 | -82,5 | 280 | seamount | sloping | rocks | high rugosity |
| 2016 | CIMAR22 | *P. bahamondei* | -24,7 | -82,5 | 280 | seamount | flat | rocks | high rugosity |
| 2016 | CIMAR22 | *P. rathbuni* | -24,7 | -82,5 | 280 | seamount | vertical | rocks | high rugosity |
| 2016 | CIMAR22 | *P. bahamondei* | -25,8 | -83,3 | 350 | island | sloping | sand | low rugosity |
| 2016 | CIMAR22 | *P. rathbuni* | -25,7 | -82,5 | 200 | seamount | flat | sand | medium rugosity |
| 2016 | CIMAR22 | *P. rathbuni* | -25,7 | -82,5 | 200 | seamount | flat | sand | medium rugosity |
| 2016 | CIMAR22 | *P. rathbuni* | -25,7 | -83 | 220 | seamount | flat | sand | low rugosity |
| 2019 | EPIC19 | *P. bahamondei* | -26,5 | -79,8 | 684 | island | sloping | rocks | high rugosity |
| 2019 | EPIC19 | *P. bahamondei* | -26,5 | -79,8 | 675 | island | sloping | rocks | high rugosity |
| 2019 | EPIC19 | *P. bahamondei* | -26,5 | -79,8 | 605 | island | flat | rocks | high rugosity |
| 2019 | EPIC19 | *P. bahamondei* | -26,5 | -79,8 | 577 | island | sloping | rocks | high rugosity |
| 2019 | EPIC19 | *P. bahamondei* | -26,5 | -79,8 | 577 | island | flat | rocks | high rugosity |
| 2019 | EPIC19 | *P. bahamondei* | -26,5 | -79,8 | 546 | island | sloping | rocks | high rugosity |
| 2019 | EPIC19 | *P. bahamondei* | -26,5 | -79,8 | 524 | island | flat | rocks | high rugosity |
| 2019 | EPIC19 | *P. bahamondei* | -26,5 | -79,8 | 524 | island | flat | rocks | high rugosity |
| 2019 | EPIC19 | *P. bahamondei* | -26,5 | -79,8 | 524 | island | flat | rocks | high rugosity |
| 2019 | EPIC19 | *P. bahamondei* | -26,5 | -79,8 | 524 | island | sloping | rocks | high rugosity |
| 2019 | EPIC19 | *P. bahamondei* | -26,5 | -79,8 | 524 | island | sloping | rocks | high rugosity |
| 2019 | EPIC19 | *P. bahamondei* | -26,5 | -79,8 | 472 | island | flat | rocks | high rugosity |
| 2019 | EPIC19 | *P. bahamondei* | -26,5 | -79,8 | 450 | island | sloping | rocks | high rugosity |
| 2019 | EPIC19 | *P. bahamondei* | -26,5 | -79,8 | 450 | island | flat | rocks | high rugosity |
| 2019 | EPIC19 | *P. bahamondei* | -26,5 | -79,8 | 418 | island | sloping | sand | low rugosity |
| 2019 | EPIC19 | *P. bahamondei* | -26,5 | -79,8 | 417 | island | sloping | rocks | low rugosity |
| 2019 | EPIC19 | *P. bahamondei* | -26,5 | -79,8 | 417 | island | sloping | rocks | low rugosity |
| 2019 | EPIC19 | *P. bahamondei* | -26,5 | -79,8 | 355 | island | flat | sand | low rugosity |
| 2019 | EPIC19 | *P. bahamondei* | -26,5 | -79,8 | 355 | island | flat | sand | low rugosity |
| 2019 | EPIC19 | *P. bahamondei* | -26,5 | -79,8 | 334 | island | sloping | sand | low rugosity |
| 2019 | EPIC19 | *P. bahamondei* | -26,5 | -79,8 | 304 | island | sloping | sand | low rugosity |
| 2019 | EPIC19 | *P. bahamondei* | -26,5 | -79,8 | 274 | island | sloping | sand | low rugosity |
| 2019 | EPIC19 | *P. bahamondei* | -26,5 | -79,8 | 274 | island | sloping | rocks | high rugosity |
| 2019 | EPIC19 | *P. rathbuni* | -26,5 | -79,8 | 250 | island | sloping | rocks | high rugosity |
| 2019 | EPIC19 | *P. rathbuni* | -26,5 | -79,8 | 250 | island | flat | sand | low rugosity |
| 2019 | EPIC19 | *P. bahamondei* | -26,5 | -79,8 | 250 | island | flat | sand | low rugosity |
| 2019 | EPIC19 | *P. bahamondei* | -26,5 | -79,8 | 250 | island | flat | sand | low rugosity |
| 2019 | EPIC19 | *P. rathbuni* | -26,5 | -79,8 | 229 | island | flat | sand | low rugosity |
| 2019 | EPIC19 | *P. rathbuni* | -26,5 | -79,8 | 229 | island | flat | sand | low rugosity |
| 2019 | EPIC19 | *P. bahamondei* | -25,4 | -81,8 | 475 | seamount | flat | rocks | high rugosity |
| 2019 | EPIC19 | *P. bahamondei* | -25,4 | -81,8 | 463 | seamount | flat | rocks | medium rugosity |
| 2019 | EPIC19 | *P. bahamondei* | -25,4 | -81,8 | 456 | seamount | flat | sand | medium rugosity |
| 2019 | EPIC19 | *P. bahamondei* | -25,4 | -81,8 | 448 | seamount | flat | rocks | medium rugosity |
| 2019 | EPIC19 | *P. bahamondei* | -25,4 | -81,8 | 430 | seamount | flat | rocks | medium rugosity |
| 2019 | EPIC19 | *P. bahamondei* | -25,4 | -81,8 | 430 | seamount | flat | rocks | medium rugosity |
| 2019 | EPIC19 | *P. bahamondei* | -25,4 | -81,8 | 404 | seamount | flat | rocks | medium rugosity |
| 2019 | EPIC19 | *P. rathbuni* | -25,4 | -81,8 | 371 | seamount | flat | rocks | medium rugosity |

**Table S3.** Historical data for the presence of *Paromola rathbuni* and *Projasus bahamondei* in the region of Nazca, Salas & Gómez and Juan Fernández ridges, Southeast Pacific. The revision included available information extracted from previous scientific cruises, fishing data, online databases and published articles. For each record, the following information was indicated: year, station, latitude, longitude, depth, taxa, subsystem type and information source. GBIF (Global Biodiversity Information Facility).

| **Historical distribution available for *Projasus bahamondei* and *Paromola rathbuni*** | | | | | | | |
| --- | --- | --- | --- | --- | --- | --- | --- |
| **Year** | **Station** | **Lat (S)** | **Lon (W)** | **Depth (m)** | **Taxa** | **Subsystem** | **Data source** |
| 1980 | HD73 | -25,7 | -85,4 | 220 | *P. rathbuni* | Seamount | USA cruise |
| 1987 | 12C | -25,7 | -85,4 | 160 | *P. rathbuni* | Seamount | Russian cruises |
| 1987 | 12G | -25,6 | -85,4 | 162 | *P. rathbuni* | Seamount | Russian cruises |
| 1987 | 12E | -25,7 | -85,4 | 165 | *P. rathbuni* | Seamount | Russian cruises |
| 1983 | 12K | -25,6 | -85,4 | 169 | *P. rathbuni* | Seamount | Russian cruises |
| 1983 | 13 A | -24,7 | -85,4 | 169 | *P. rathbuni* | Seamount | Russian cruises |
| 1980 | 12H | -25,6 | -85,4 | 175 | *P. rathbuni* | Seamount | Russian cruises |
| 1980 | 12F | -25,6 | -85,4 | 180 | *P. rathbuni* | Seamount | Russian cruises |
| 1980 | 12J | -25,6 | -85,4 | 190 | *P. rathbuni* | Seamount | Russian cruises |
| 1980 | 12L | -25,6 | -85,5 | 190 | *P. rathbuni* | Seamount | Russian cruises |
| 1980 | 12M | -25,6 | -85,5 | 215 | *P. rathbuni* | Seamount | Russian cruises |
| 1980 | 12 A | -25,6 | -85,3 | 220 | *P. rathbuni* | Seamount | Russian cruises |
| 1987 | 12D | -25,7 | -85,4 | 220 | *P. rathbuni* | Seamount | Russian cruises |
| 1979 | 12I | -25,7 | -85,4 | 225 | *P. rathbuni* | Seamount | Russian cruises |
| 1980 | 12B | -25,7 | -85,4 | 235 | *P. rathbuni* | Seamount | Russian cruises |
| 1987 | 12N | -25,6 | -85,5 | 242 | *P. rathbuni* | Seamount | Russian cruises |
| 1987 | 12 O | -25,6 | -85,5 | 255 | *P. rathbuni* | Seamount | Russian cruises |
| 1987 | 15B | -25,9 | -84,4 | 280 | *P. rathbuni* | Seamount | Russian cruises |
| 1987 | 12P | -25,6 | -85,4 | 280 | *P. rathbuni* | Seamount | Russian cruises |
| 1987 | 15 A | -25,9 | -84,4 | 285 | *P. rathbuni* | Seamount | Russian cruises |
| 1983 | 18R | -22,1 | -81,3 | 300 | *P. rathbuni* | Seamount | Russian cruises |
| 1983 | 19 | -21,5 | -81,7 | 300 | *P. rathbuni* | Seamount | Russian cruises |
| 1980 | 13B | -24,8 | -85,5 | 320 | *P. rathbuni* | Seamount | Russian cruises |
| 1980 | 16 | -23,9 | -84,7 | 295 | *P. rathbuni* | Seamount | Russian cruises |
| 1983 | 17R | -23,5 | -83,3 | 300 | *P. bahamondei* | Seamount | Russian cruises |
| 1983 | 18R | -22,1 | -81,3 | 300 | *P. bahamondei* | Seamount | Russian cruises |
| 1983 | 19 | -21,5 | -81,7 | 300 | *P. bahamondei* | Seamount | Russian cruises |
| 2000 | T1 | -33,6 | -78,8 | 100 | *P. rathbuni* | Island | Arana (2000) |
| 2000 | 12 | -33,6 | -78,9 | 100 | *P. rathbuni* | Island | Arana (2000) |
| 2000 | T2 | -33,6 | -78,9 | 100 | *P. rathbuni* | Island | Arana (2000) |
| 2000 | T3 | -33,7 | -78,9 | 100 | *P. rathbuni* | Island | Arana (2000) |
| 2000 | T1 | -33,6 | -78,8 | 200 | *P. rathbuni* | Island | Arana (2000) |
| 2000 | T2 | -33,5 | -78,9 | 200 | *P. rathbuni* | Island | Arana (2000) |
| 2000 | T2 | -33,6 | -78,9 | 200 | *P. rathbuni* | Island | Arana (2000) |
| 2000 | T2 | -33,6 | -78,9 | 200 | *P. rathbuni* | Island | Arana (2000) |
| 2000 | T3 | -33,7 | -79,0 | 200 | *P. rathbuni* | Island | Arana (2000) |
| 2000 | T3 | -33,6 | -79,0 | 200 | *P. rathbuni* | Island | Arana (2000) |
| 2000 | T4 | -33,7 | -78,8 | 200 | *P. rathbuni* | Island | Arana (2000) |
| 2000 | T4 | -33,7 | -78,8 | 250 | *P. rathbuni* | Island | Arana (2000) |
| 1985 | NA | -25,3 | -85,1 | 280 | *P. rathbuni* | Seamount | Arana (2000) |
| 2000 | T1 | -33,6 | -78,8 | 300 | *P. rathbuni* | Island | Arana (2000) |
| 2000 | T1 | -33,6 | -78,8 | 300 | *P. rathbuni* | Island | Arana (2000) |
| 2000 | T2 | -33,5 | -79,0 | 300 | *P. rathbuni* | Island | Arana (2000) |
| 2000 | T3 | -33,6 | -79,0 | 300 | *P. rathbuni* | Island | Arana (2000) |
| 2000 | T4 | -33,7 | -78,8 | 300 | *P. rathbuni* | Island | Arana (2000) |
| 2000 | T4 | -33,7 | -78,8 | 300 | *P. rathbuni* | Island | Arana (2000) |
| 2009 | NA | -33,5 | -78,8 | 350 | *P. rathbuni* | Island | Ahumada & Arana (2009) |
| 2009 | NA | -33,6 | -78,8 | 350 | *P. rathbuni* | Island | Ahumada & Arana (2009) |
| 2009 | NA | -33,7 | -78,8 | 350 | *P. rathbuni* | Island | Ahumada & Arana (2009) |
| 2000 | T3 | -33,6 | -79,0 | 350 | *P. rathbuni* | Island | Arana (2000) |
| 2000 | T3 | -33,7 | -79,0 | 350 | *P. rathbuni* | Island | Arana (2000) |
| NA | NA | -26,4 | -79,8 | 175 | *P. bahamondei* | Island | Arana (2014) |
| 1976 | NA | -35,3 | -72,9 | 260 | *P. bahamondei* | Continental ridge | Andrade & Báez (1980) |
| 1990 | NA | -20,8 | -80,9 | 320 | *P. bahamondei* | Seamount | Poupin (2003) |
| 1990 | NA | -21,4 | -81,6 | 320 | *P. bahamondei* | Seamount | Poupin (2003) |
| 1976 | NA | -30,0 | NA | 350 | *P. bahamondei* | Continental ridge | Andrade & Báez (1980) |
| 2004 | NA | -32,9 | -73,9 | 450 | *P. bahamondei* | seamount | Cañete (2012) |
| 1976 | NA | -35,0 | -71,2 | 500 | *P. bahamondei* | Continental ridge | Andrade & Báez (1980) |
| NA | NA | -39,9 | -73,9 | NA | *P. bahamondei* | seamount | Arana (2014) |
| NA | NA | -28,5 | NA | NA | *P. bahamondei* | Continental ridge | Arana (2014) |
| NA | NA | -35,3 | NA | NA | *P. bahamondei* | Continental ridge | Arana (2014) |
| 1973 | NA | -25,8 | -83,3 | 100 | *P. rathbuni* | Island | GBIF Online Database |
| 1973 | NA | -25,8 | -83,3 | 100 | *P. rathbuni* | Island | GBIF Online Database |
| 1973 | NA | -26,5 | -79,9 | 215 | *P. rathbuni* | Island | GBIF Online Database |
| 1973 | NA | -26,5 | -79,9 | 215 | *P. rathbuni* | Island | GBIF Online Database |
| 1973 | NA | -26,5 | -79,9 | 215 | *P. rathbuni* | Island | GBIF Online Database |
| 1973 | NA | -25,4 | -81,8 | 250 | *P. rathbuni* | Seamount | GBIF Online Database |
| 1973 | NA | -25,4 | -81,8 | 250 | *P. rathbuni* | Seamount | GBIF Online Database |
| 1973 | NA | -25,4 | -81,8 | 250 | *P. rathbuni* | Seamount | GBIF Online Database |
| 1973 | NA | -25,4 | -81,8 | 250 | *P. rathbuni* | Seamount | GBIF Online Database |
| 1973 | NA | -26,3 | -80,0 | 350 | *P. rathbuni* | Seamount | GBIF Online Database |
| 1973 | NA | -33,6 | -78,8 | NA | *P. rathbuni* | Island | GBIF Online Database |
| 1973 | NA | -33,6 | -78,8 | NA | *P. rathbuni* | Island | GBIF Online Database |
| 1973 | NA | -33,6 | -78,8 | NA | *P. rathbuni* | Island | GBIF Online Database |
| 1982 | NA | -21,2 | -90,4 | 113 | *P. bahamondei* | Seamount | GBIF Online Database |
| NA | NA | -26,4 | -79,8 | 175 | *P. bahamondei* | Island | GBIF Online Database |
| 2016 | NA | -26,3 | -80,0 | 175 | *P. bahamondei* | Island | GBIF Online Database |
| 2016 | NA | -26,3 | -80,0 | 175 | *P. bahamondei* | Island | GBIF Online Database |
| 2016 | NA | -26,3 | -80,0 | 175 | *P. bahamondei* | Island | GBIF Online Database |
| 1980 | NA | -22,2 | -81,4 | 225 | *P. bahamondei* | Seamount | GBIF Online Database |
| 1980 | NA | -22,2 | -81,4 | 227 | *P. bahamondei* | Seamount | GBIF Online Database |
| 1980 | NA | -22,2 | -81,3 | 230 | *P. bahamondei* | Seamount | GBIF Online Database |
| 1987 | NA | -22,1 | -81,3 | 233 | *P. bahamondei* | Seamount | GBIF Online Database |
| 1987 | NA | -22,1 | -81,3 | 242 | *P. bahamondei* | Seamount | GBIF Online Database |
| 1976 | NA | -35,3 | -72,8 | 260 | *P. bahamondei* | Continental ridge | GBIF Online Database |
| 1987 | NA | -22,1 | -81,3 | 267 | *P. bahamondei* | Seamount | GBIF Online Database |
| 1980 | NA | -23,9 | -84,7 | 295 | *P. bahamondei* | Seamount | GBIF Online Database |
| 1979 | NA | -32,6 | -71,6 | 300 | *P. bahamondei* | Continental ridge | GBIF Online Database |
| 1987 | NA | -20,8 | -80,9 | 316 | *P. bahamondei* | Seamount | GBIF Online Database |
| 1980 | NA | -21,5 | -81,6 | 325 | *P. bahamondei* | Seamount | GBIF Online Database |
| 1980 | NA | -21,4 | -81,5 | 330 | *P. bahamondei* | Seamount | GBIF Online Database |
| 1987 | NA | -21,4 | -81,7 | 330 | *P. bahamondei* | Seamount | GBIF Online Database |
| 1980 | NA | -21,5 | -81,7 | 330 | *P. bahamondei* | Seamount | GBIF Online Database |
| 1987 | NA | -21,5 | -81,6 | 336 | *P. bahamondei* | Seamount | GBIF Online Database |
| 1987 | NA | -31,9 | -71,6 | 350 | *P. bahamondei* | Seamount | GBIF Online Database |
| 1979 | NA | -22,2 | -81,4 | 355 | *P. bahamondei* | Continental ridge | GBIF Online Database |
| NA | NA | -32,1 | -71,6 | 360 | *P. bahamondei* | Seamount | GBIF Online Database |
| 1977 | NA | -20,8 | -80,9 | 370 | *P. bahamondei* | Continental ridge | GBIF Online Database |
| 1987 | NA | -36,6 | -72,0 | 390 | *P. bahamondei* | Seamount | GBIF Online Database |
| 2021 | NA | -32,5 | -71,6 | 400 | *P. bahamondei* | Continental ridge | GBIF Online Database |
| 1977 | NA | -32,1 | -71,6 | 400 | *P. bahamondei* | Continental ridge | GBIF Online Database |
| 1977 | NA | -32,7 | -71,6 | 400 | *P. bahamondei* | Continental ridge | GBIF Online Database |
| 1976 | NA | -31,9 | -71,6 | 400 | *P. bahamondei* | Continental ridge | GBIF Online Database |
| 1976 | NA | -32,5 | -71,6 | 400 | *P. bahamondei* | Continental ridge | GBIF Online Database |
| 1976 | NA | -31,9 | -71,6 | 400 | *P. bahamondei* | Continental ridge | GBIF Online Database |
| 1976 | NA | -32,5 | -71,6 | 420 | *P. bahamondei* | Continental ridge | GBIF Online Database |
| 1980 | NA | -31,9 | -71,6 | 420 | *P. bahamondei* | Continental ridge | GBIF Online Database |
| 1979 | NA | -21,4 | -81,7 | 420 | *P. bahamondei* | Continental ridge | GBIF Online Database |
| 1987 | NA | -30,0 | -71,5 | 450 | *P. bahamondei* | Seamount | GBIF Online Database |
| 1981 | NA | -23,4 | -83,3 | 475 | *P. bahamondei* | Continental ridge | GBIF Online Database |
| 1980 | NA | -21,4 | -81,6 | 515 | *P. bahamondei* | Seamount | GBIF Online Database |
| 1987 | NA | -21,4 | -81,6 | 515 | *P. bahamondei* | Seamount | GBIF Online Database |
| NA | NA | -26,4 | -79,8 | NA | *P. bahamondei* | Island | GBIF Online Database |
| 2016 | SF9 | -25,8 | -83,3 | 200 | *P. rathbuni* | Seamount | CIMAR-22 |
| 2016 | SF8 | -25,7 | -83,0 | 220 | *P. rathbuni* | Seamount | CIMAR-22 |
| 2016 | SF2 | -24,7 | -82,5 | 280 | *P. rathbuni* | Seamount | CIMAR-22 |
| 2016 | S22 | -25,8 | -83,3 | 350 | *P. rathbuni* | Island | CIMAR-22 |
| 2019 | 3 A1 | -26,5 | -79,8 | 210 | *P. rathbuni* | Island | EPIC-19 |
| 2019 | 4 A2 | -25,4 | -81,8 | 234 | *P. rathbuni* | Seamount | EPIC-19 |
| 2019 | 4 DT | -25,4 | -81,8 | 234 | *P. rathbuni* | Seamount | EPIC-19 |
| 2019 | 4 A1 | -25,4 | -81,8 | 530 | *P. rathbuni* | Seamount | EPIC-19 |
| 2019 | 3 DT | -26,5 | -79,8 | 420 | *P. bahamondei* | Island | EPIC-19 |
| 2019 | 4 DT | -25,4 | -81,8 | 415 | *P. bahamondei* | Seamount | EPIC-19 |

Table S4. Correlation matrix for abiotic variables, including physico-chemical (*in situ* Temperature, Dissolved Oxygen-O_2_, Salinity) and geographic/geologic (Longitude W, Latitude S, Depth) variables. The correlation matrix for *Paromola rathbuni* and *Projasus bahamondei* data sets are shown separately*.* Bi-variate high correlation (r> 0.50) is indicated in bold.

|  | ***Paromola rathbuni*** | | | | | | ***Projasus bahamondei*** | | | | | |
| --- | --- | --- | --- | --- | --- | --- | --- | --- | --- | --- | --- | --- |
|  | **Salinity** | **O2** | **Temperature** | **Latitude** | **Longitude** | **Depth** | **Salinity** | **O2** | **Temperature** | **Latitude** | **Longitude** | **Depth** |
| **Salinity** | 1.0000 | 0.1020 | **0.5258** |  |  |  | 1.000 | 0.0091 | **0.5833** |  |  |  |
| **O2** | 0.1020 | 1.0000 | 0.4847 |  |  |  | 0.0091 | 1.0000 | 0.2821 |  |  |  |
| **Temperature** | **0.5258** | 0.4847 | 1.0000 |  |  |  | **0.5833** | 0.2821 | 1.0000 |  |  |  |
| **Latitude** |  |  |  | 1.0000 | -0.9256 | 0.1472 |  |  |  | 1.0000 | -0.1117 | -0.0276 |
| **Longitude** |  |  |  | -0.9256 | 1.0000 | -0.4707 |  |  |  | -0.1117 | 1.0000 | 0.3034 |
| **Depth** |  |  |  | 0.1472 | -0.4707 | 1.0000 |  |  |  | -0.0276 | 0.3034 | 1.000 |

**Reference List**

Andrade, H. & Báez, P. (1980) Crustáceos Decápodos asociados a la pesquería de *Heterocarpus reedi* Bahamonde, 1955, en la zona central de Chile. Boletín del Museo Nacional de Historia Natural, 37, 261-267. <https://doi.org/10.54830/bmnhn.v37.1980.490>

Arana, P. (2000). Pesca exploratoria con trampas alrededor de las islas Robinson Crusoe y Santa Clara, Archipiélago de Juan Fernández, Chile. Investigaciones Marinas, 28, 39–52.<https://doi.org/10.4067/s0717-71782000002800005>

Ahumada, M. & Arana, P. (2009). Pesca artesanal de Cangrejo dorado (*Chaceon chilensis*) en el Archipiélago de Juan Fernández, Chile. Latin American Journal of Aquatic Research, 37(3): 285-296. DOI: 10.3856/vol37-issue3-fulltext-2

Arana, P. (2014). Chilean jagged lobster, *Projasus bahamondei*, in the southeastern Pacific Ocean: current state of knowledge. Latin American Journal of Aquatic Research, 42(1), 1–17.<https://doi.org/10.3856/vol42-issue1-fulltext-1>

Poupin, J. (2003). Crustacea Decapoda and Stomatopoda of Easter Island and surrounding areas. A documented checklist with historical overview and biogeographic comments. Atoll Research Bulletin. 500, 1–50.<https://doi.org/10.5479/si.00775630.500.1>

**Electronic References**

*Projasus bahamondei* George*, 1976*: GBIF.org (27 April 2022) GBIF Occurrence Download <https://doi.org/10.15468/dl.3ax4k2>

*Paromola rathbuni Porter, 1908*: GBIF.org (24 April 2022) GBIF Occurrence Download <https://doi.org/10.15468/dl.k9rgr8>
